# High-Dose Radioiodine Outpatient Treatment: An Initial Experience in Thailand

**Published:** 2015

**Authors:** Danupon Nantajit, Sureerat Saengsuda, Pattama NaNakorn, Yuthana Saengsuda

**Affiliations:** 1 Office of Atoms for Peace, Bangkok 10900, Thailand; 2 Chulabhorn Hospital, Bangkok 10210, Thailand; 3 Rajavithi Hospital, Bangkok 10400, Thailand; 4 College of Medicine, Rangsit University, Bangkok 10400, Thailand

**Keywords:** Radiation exposure, Radiation protection, Radioactive iodine, Throid carcinoma

## Abstract

**Objective(s):**

The aim of this study was to determine whether high-dose radioactive iodine (Na^131^I) outpatient treatment of patients with thyroid carcinoma is a pragmatically safe approach, particularly for the safety of caregivers.

**Methods:**

A total of 79 patients completed the radiation-safety questionnaires prior to receiving high-dose radioactive iodine treatment. The questionnaire studied the subjects’ willingness to be treated as outpatients, along with the radiation safety status of their caregivers and family members. In patients, who were selected to be treated as outpatients, both internal and external radiation exposures of their primary caregivers were measured, using thyroid uptake system and electronic dosimeter, respectively.

**Results:**

Overall, 62 out of 79 patients were willing to be treated as outpatients; however, only 44 cases were eligible for the treatment. The primary reason was that the patients did not use exclusive, separated bathrooms. The caregivers of 10 subjects, treated as outpatients, received an average radiation dose of 138.1 microsievert (mSv), which was almost entirely from external exposure; the internal radiation exposures were mostly at negligible values. Therefore, radiation exposure to caregivers was significantly below the public exposure limit (1 mSv) and the recommended limit for caregivers (5 mSv).

**Conclusion:**

A safe ^131^I outpatient treatment in patients with thyroid carcinoma could be achieved by selective screening and providing instructions for patients and their caregivers.

## Introduction

The United States Nuclear Regulatory Commission (USNRC) revised Title 10 of the Code of Federal Regulations (10CFR 35.75) in 1997, allowing the release of patients immediately after high-dose radioiodine (^131^I) treatment. Moreover, the International Commission on Radiological Protection (ICRP) suggested reasonable limits of radiation exposure a person may receive from a treated patient, which made high-dose ^131^I outpatient treatment an alternative mode of therapy (1).

Typically, patients who are treated with radioactive iodine-131 (above 30 mCi or 1.1 GBq) are subjected to confinement in the medical facility until radiation exposure rates from their bodies become less than 43.5 μSv/h at a 1-meter distance (12.9×10^−7^ C/kg/h, 5 mR/h) or until the patients’ radionuclide activities become less than 1.1 GBq (30 mCi) (2). Therefore, patients who are treated with ^131^I at a dose higher than 1.1 GBq (30 mCi) are subjected to the confinement in the hospital for a period of time before being discharged.

Changes in regulations from an activity-based limit to a dose-based limit allow patient release if radiation exposure from the patient is likely to result in a total effective dose equivalent of less than 5.0 mSv for any maximally-exposed individuals. In principle, the new regulation was supported by the ICRP publication 94, indicating that the release criteria in certain countries are overly restrictive and could be more focused on radiation protection without appropriate justification or optimization (3).

The Safety Reports Series No. 63 by The International Atomic Energy Agency (IAEA) also stated that an exposure of a few millisieverts could be tolerated by family members, with the exception of pregnant women and children, as there are direct effects on both patients and those who care for them (4).

The limited number of beds, nursing staff, and facilities and the growing number of population and patients, especially in developing countries, have been major issues for public healthcare. Each patient receiving ^131^I treatment for thyroid cancer requires approximately 2-3 days of hospital stay as the standard practice (5-6). At many public hospitals in Thailand, patients may need to wait for several months for their treatment, considering the number of beds available for nuclear medicine patients at the hospitals.

In outpatient treatment, several factors including patient environment, financial costs, waste disposal, and psychological effects contribute to patient release (4). Nevertheless, one of the primary issues which radioiodine-treated patients and their relatives are concerned about is radiation safety. Many eligible patients request to stay at hospitals in order to minimize radiation exposure to their family members; the eligibility criteria include living in a suitable environment, self-care abilities, and the possibility of being treated as an outpatient for radioiodine therapy.

Currently, the incidence of thyroid cancer in Thailand is at 2.2:100,000 population, with incidence rates of 1.1:100,000 and 3.2:100,000 in males and females, respectively (7). In fact, the release of patients, meeting the criteria for radiation safety, would reduce load of hospitals, specifically public hospitals, and would benefit other patients requiring hospitalization.

As recent evidence suggests, only after one day of hospitalization, most patients receiving up to 7.4 GBq (200 mCi) of ^131^I already have permissible exposure rates below 70 μSvh^-1^ since over 75-80% of the dosage is excreted through urination within 24 hours of ^131^I administration (4, 8). Thus, the standard practice of restraining patients in hospitals for 2-3 days should not be mandatory for most cases, as a fraction of radioactivity has decayed and most of the ingested activity has already been excreted through urination after one day.

In 2004, Nuclear Medicine Society of Thailand issued a statement indicating that high-dose ^131^I (3.7-7.4 GBq) outpatient treatment of patients with thyroid disease is an admissible practice. Patients contemplating such an option should be well-informed about the risks and thoroughly assess their conditions and environments.

Certain guidelines have been developed for outpatient treatment; however, they have not been widely accepted by physicians and are rarely adopted for patients due to radiation safety concerns. The Office of Atoms for Peace (OAP), as the national regulator of nuclear applications, also voiced its concern regarding public confidence.

Therefore, in this study, in order to explore the possibility of implementing these guidelines and gain acceptance by physicians and the public, the radiation exposure of the caregivers of patients, who underwent high-dose (≥ 3.7 GBq) ^131^I treatment, was evaluated. Instructions and guidelines for radiation safety were given to the patients and caregivers to minimize potential exposures. Both external and internal radiation doses were monitored to determine whether outpatient treatment with high-dose ^131^I is an acceptable practice in Thai population.

## Methods

### Patients and patient selection

Patients with differentiated thyroid carcinoma, treated at Rajavithi Hospital in Bangkok, Thailand, who required ^131^I treatment, were screened for outpatient treatment. Overall, 79 patients, who met the inclusion criteria, were asked to complete the questionnaires; the answers were evaluated to assess their suitability for outpatient treatment.

The questionnaire mainly focused on the following points:

1- Patients: gender, age, self-care ability, literacy level, willingness to be treated as an outpatient, pregnancy status, type of thyroid disease, amount of required ^131^I, urinary incontinence, swallowing and vomiting conditions, medical needs, and hospital stay requirements.

2- Caregivers: gender, age, and pregnancy status.

3- Radiation safety: number of family members in the household, age of family members in the household, patient’s ability to avoid close contact with pregnant women and children for 7 days, the possibility of the relocation of children for 5 days after the treatment, number of bathrooms (with shower and toilet) in the house, having a separate bathroom, mode of transport and accompaniment during the transport, presence of caregivers during the transport, pregnancy plan within 6 months after the treatment, if the patient can sleep alone, if the patient can keep a distance of 2 meters away from others, if the patient can eat alone for 7 days, and if the patient can stay at home for 3 days.

The questionnaire used in this study is provided in Supplementary Document 1.

### Criteria for outpatient selection

The questionnaires were evaluated by a radiation safety officer (RSO) in consultation with the patients’ nuclear medicine physicians to assess any potential radiation exposures to family members and the public. Qualified prospective patients were further interviewed by the RSO about their conditions for the outpatient treatment. Detailed arrangements for radiation safety were planned by the RSO according to each patient’s characteristics and conditions.

### Guidelines and instructions given to patients

The instructions given to patients were developed using practice recommendations of the American Thyroid Association (9-10). Radiation safety instructions for patients and caregivers after ^131^I treatment are provided in Supplementary Document 2.

### Radioactive ^131^I treatment

Patients with differentiated thyroid carcinoma were given oral radioactive ^131^I in form of sodium iodine, orally for therapy, with a planned activity range of 3.7–5.55 GBq (100–150 mCi), according to physicians’ prescription. Patients were selected for outpatient treatment, based on their radiation safety status, evaluated by the RSO. Patients were also inquired for their voluntary participation, and patient consent forms were obtained.

### Radiation exposure measurements

Patients and their caregivers were instructed regarding radiation protection guidelines. The caregivers were given personal electronic dosimeters (RAD-60S personal dosimeter, Mirion Technologies (RADOS) Oy, Turku, Finland) to monitor the patients’ external radiation exposure for one week since the first day of patient treatment.

The caregivers were instructed to always wear the dosimeters on the torso at home and leave them in their bedrooms away from the patients as they leave the house. For internal dosimetry, caregivers were scanned for thyroid ^131^I activity for 10 minutes each time, prior to and 7 days after patient treatments, using thyroid uptake system (Captus 3000) and bioassay measurement protocol according to the manufacturer’s instructions (Capintec Inc., Ramsey, New Jersey, USA). Total radiation doses of caregivers were determined using the sum of external and internal radiation doses.

## Results

As the data analysis indicated, of 79 patients, 62 cases (78.5%) were willing to be treated as outpatients, acknowledging that the risk of radiation to their relatives would be minimal if they were to strictly follow the radiation safety instructions. After evaluating the answers to the questionnaire (Table 1), 44 patients (55.7%) were found to be suitable for outpatient treatment.

**Table 1:** Main factors influencing the decision of high-dose radioactive iodine outpatient treatment in 79 patients

No.	Factor	Number of Patients	Percentage
1	Willingness to be treated as an outpatient	62	78.5
2	No separate bathroom for patients	37	46.8
3	Duration of journey home over 3 hours	17	21.5
4	Illiteracy	12	15.2
5	Required hospitalization	9	11.4
6	Incontinence problem	4	5.1
7	Cannot avoid contact with children at home	4	5.1

The inclusion criterion was patient’s willingness to participate in the study, while the main exclusion criteria were as follows: 1) a long time required for travelling home; 2) urinary incontinence; 3) not having separate bedroom or bathroom for patient’s exclusive use; and 4) having children at home.

The primary criterion, which most patients failed to meet, was that the patients did not have exclusive bathrooms for their use (46.8%). Also, in 21.5% of the patients, it took more than 3 hours to travel back home from the hospital, which forbade them from being treated as outpatients, since the driver (or any person within the same vehicle) would be potentially exposed to a substantial amount of radiation. In addition, the patients would require using public bathrooms, which could cause radiation exposure to the public.

Illiteracy was also reported in 15.2% of the cases, which made it difficult for the patients to understand and follow the radiation safety instructions. As previously mentioned, other exclusion criteria were having children at home and urinary incontinence (or other medical conditions), requiring hospitalization. These factors impeded the patients from receiving outpatient treatments, as shown in Table 1.

After evaluating the questionnaires and interviewing prospective outpatients, we selected eligible patients with at least one family member, who could act as a caregiver. Demographic characteristics of the patients and their corresponding caregivers are indicated in Table 2.

**Table 2:** Patient and caregiver demographic characteristics

No.	Patients		Caregivers	
	Gender	Age	Gender	Age
1	F	79	M	80
2	F	33	M	64
3	F	38	M	39
4	F	54	M	57
5	F	50	M	50
6	F	81	F	60
7	F	65	M	61
8	F	24	F	54
9	F	78	F	46
10	F	46	M	45

As thyroid carcinoma is more prevalent among females (11), the selected patients (n=10) were all females, while the caregivers were their spouses, siblings, or children. The caregivers were evaluated for their background thyroid activity prior to the outpatient treatment.

Personal electronic dosimeters were given to the caregivers and they were instructed to wear them whenever possible. External radiation doses, which were recorded by the electronic dosimeter for 7 days, showed that the caregivers received a dose range of 21-672 μSv (average=138.1 μSv), which neither exceeded the recommended dose limit to caregivers (5 mSv) nor the annual limit for public (1 mSv), set by the USNRC.

As shown in Table 3, the primary factor affecting the received dosage by the caregivers was the amount of time spent with the patient within the vehicle while returning home. Caregivers, who had received more than 100 μSv, had travelled home with the patients and had spent a substantial amount of time with them.

**Table 3:** Radiation doses received by the caregivers including the approximate time spent in the vehicle with patients

No.	^131^I administered (GBq)	External dose (μSv)	Internal dose (μSv)	Total dose received (μSv)	Approximate time spent in the vehicle (Min)
1	5.55	24	Neg	24	0
2	5.55	21	Neg	21	0
3	5.92	62	Neg	62	60
4	5.77	27	Neg	27	0
5	5.85	130	Neg	130	45
6	5.81	32	Neg	32	10
7	6.11	187	Neg	187	90
8	3.89	66	Neg	66	0
9	5.81	160	0.1	160.1	45
10	3.92	672	Neg	672	150
Average	5.42	138.1	-	138.1	-
S.D.	0.81	197.0	-	197.0	-
S.E.	-	62.3	-	62.3	-

Neg.: Negligible value 0– Patient and caregiver returned home in separate vehicles

0– Patient and caregiver returned home in separate vehicles

One of the intriguing results of this study was that the internal doses received by the caregivers were insignificant for all the subjects. Only one caregiver received an approximate dose of 0.1 μSv, which was insignificant, compared to the external radiation exposure. Expectedly, if the caregivers and patients strictly follow the given instructions, internal exposures to the caregivers will be marginalized.

## Discussion

The gathered results indicated that a substantial number of patients required no hospitalization, i.e., more than half of the patients, hospitalized after ^131^I therapy, could use home care.

The radiation exposure results were consistent with a previous report, indicating that one-third of the caregiver dose was received during the transport from the hospital (12). In this study, the most significant contributing factor was the journey home from the hospital. In fact, the dose received during the stay at home was much less, especially for caregiver number 10, who needed to spend over two hours with the patient within the same vehicle.

These results are also in agreement with a previous study, which showed that the cumulative dose to the nursing staff after ^131^I treatment was dependent on patient mobility; this value was estimated at 0.08 mSv for a self-caring patient and 6.3 mSv for a totally helpless patient (13). However, the amount of radioactive ^131^I administered to the patients appeared to have little influence on the radiation doses received by the caregivers.

Compared to previous studies, which reported the radiation doses to family members (received from ^131^I therapy), our results showed that the received doses were significantly lower than those previously reported. The lower amount of received dose was probably related to both patient selection and the instructions given to the patients and caregivers.

Additionally, we inquired the caregivers regarding the amount of time they spent with the patients and learned that as the patients were capable of self-care, the time that the caregivers needed to be in their proximity was fairly limited. Most of the caregivers also went to work during the day, thus, having little chance for radiation exposure, both internally and externally. Therefore, the estimated occupancy factor between the patients and caregivers was much lower than 1.

Additionally, a previously conducted study reported that out of 26 individuals, living with patients treated with high-dose ^131^I, 25 had received a dose of less than 1 mSv, while others had received a dose of 2.8 mSv (14); these findings are in consistence with the current results. A previous study also indicated that family members of the patients received a dose range of 0.01-1.09 mSv (mean=0.24 mSv) (2).

These results indicated that outpatient ^131^I treatment can be a safe option as the estimated values of the accumulated dose (at a 1-m distance from the patient) are generally less than 5 mSv, even if the caregiver attends the patient at all times (an occupancy factor of 1). Typically, for ^131^I treatment, external exposure accounts for more than 90% of total exposure (15).

In Thailand, the patient release regulation, which mandated a 3-day hospitalization as the standard practice, was theoretically derived from a simple calculation without considering radiation attenuation of the body and other factors; however, the calculated doses were found to be inaccurate as the measured doses were significantly lower than the calculated ones (16). In fact, some patients may have retained radioactivity less than the release limit within 2 days after the treatment (17).

Moreover, theoretic doses may overestimate the dose received by an individual. For instance, for ^131^I-Tositumomab, the measured dose rate was at 60% of the theoretic dose rate, and dosimetric data estimated the mean dose received by the maximally exposed individual to be 3.06 mSv (range= 1.95–4.96 mSv), which remained within the recommended dose limit to the caregiver (18).

## Conclusion

Outpatient treatment with high-dose radioactive ^131^I is the standard practice in several countries; however, the issue of public concern has been repeatedly raised. A number of reports have measured and determined doses to individuals surrounding the patients treated with high-dose radioactive iodine (≥ 3.7 GBq) and found that outpatient treatment is a safe practice, especially if the patients and the surrounding individuals are provided with proper instructions and guidelines.

In this study, we showed that outpatient treatment is a safe and tenable alternative in Thailand. The radiation doses that the caregivers received when traveling with the patients were minimal as they followed the given instructions. Therefore, it is recommended that hospitals with limited resources (e.g., beds and nursing staff in their nuclear medicine department), should consider outpatient ^131^I treatment for those eligible patients who agree to be treated as outpatients and are able to follow radiation safety instructions.

## References

[1] (1997). United States. Dept of Energy. Office of Scientific and Technical Information. Regulatory analysis on criteria for the release of patients administered radioactive material. Final report. www.osti.gov/scitech/servlets/purl/453778.

[2] Grigsby PW, Siegel BA, Baker S, Eichling JO (2000). Radiation exposure from outpatient radioactive iodine (131I) therapy for thyroid carcinoma. JAMA.

[3] Protection (2004). ICoR. Release of patients after therapy with unsealed radionuclides. Ann ICRP.

[4] (2009). International Commission on Radiological Protection., International Atomic Energy Agency. Release of patients after radionuclide therapy.

[5] Pacilio M, Bianciardi L, Panichelli V, Argiro G, Cipriani C (2005). Management of 131I therapy for thyroid cancer: cumulative dose from in-patients, discharge planning and personnel requirements. Nucl Med Commun.

[6] Remy H, Coulot J, Borget I, Ricard M, Guilabert N, Lavielle F (2012). Thyroid cancer patients treated with 131I: radiation dose to relatives after discharge from the hospital. Thyroid.

[7] (2009). International Atomic Energy Agency. Nuclear medicine in thyroid cancer management : a practical approach.

[8] Azizmohammadi Z, Tabei F, Shafiei B, Babaei AA, Jukandan SM, Naghshine R (2013). A study of the time of hospital discharge of differentiated thyroid cancer patients after receiving iodine-131 for thyroid remnant ablation treatment. Hell J Nucl Med.

[9] Sisson JC, Freitas J, McDougall IR, Dauer LT, Hurley JR, Brierley JD (2011). Radiation safety in the treatment of patients with thyroid diseases by radioiodine 131I : practice recommendations of the American Thyroid Association. Thyroid.

[10] Hennessey JV, Parker JA, Kennedy R, Garber JR (2012). Comments regarding Practice Recommendations of the American Thyroid Association for radiation safety in the treatment of thyroid disease with radioiodine. Thyroid.

[11] Gilliland FD, Hunt WC, Morris DM, Key CR (1997). Prognostic factors for thyroid carcinoma. A population-based study of 15,698 cases from the Surveillance, Epidemiology and End Results (SEER) program 1973-1991. Cancer.

[12] Marriott CJ, Webber CE, Gulenchyn KY (2007). Radiation exposure for 'caregivers'during high-dose outpatient radioiodine therapy. Radiat Prot Dosimetry.

[13] Barrington SF, Kettle AG, O'Doherty MJ, Wells CP, Somer EJ, Coakley AJ (1996). Radiation dose rates from patients receiving iodine-131 therapy for carcinoma of the thyroid. Eur J Nucl Med.

[14] de Carvalho JW, Sapienza M, Ono C, Watanabe T, Guimaraes MI, Gutterres R (2009). Could the treatment of differentiated thyroid carcinoma with 3.7 and 5.55 GBq of (131I)NaI, on an outpatient basis, be safe?. Nucl Med Commun.

[15] Venencia CD, Germanier AG, Bustos SR, Giovannini AA, Wyse EP (2002). Hospital discharge of patients with thyroid carcinoma treated with 131I. J Nucl Med.

[16] Leslie WD, Havelock J, Palser R, Abrams DN (2002). Large-body radiation doses following radioiodine therapy. Nucl Med Commun.

[17] Culver CM, Dworkin HJ (1992). Radiation safety considerations for post-iodine-131 thyroid cancer therapy. J Nucl Med.

[18] Siegel JA, Kroll S, Regan D, Kaminski MS, Wahl RL (2002). A practical methodology for patient release after tositumomab and (131)I-tositumomab therapy. J Nucl Med.

